# Clinical evaluation of an antiinflammatory and antioxidant diet effect in 30 dogs affected by chronic otitis externa: preliminary results

**DOI:** 10.1007/s11259-015-9651-4

**Published:** 2016-01-07

**Authors:** Alessandro Di Cerbo, Sara Centenaro, Francesca Beribè, Fulvio Laus, Matteo Cerquetella, Andrea Spaterna, Gianandrea Guidetti, Sergio Canello, Giuseppe Terrazzano

**Affiliations:** School of Specialization in Clinical Biochemistry, “G. d’Annunzio” University, Chieti, Italy; Research and Development Department, SANYpet S.p.a., Bagnoli di Sopra, Padua, Italy; School of Biosciences and Veterinary Medicine, University of Camerino, Camerino, Italy; Department of Science, University of Basilicata, Potenza, Italy; Department of Translational Medical Sciences, University of Naples Federico II, Naples, Italy

**Keywords:** Nutraceutical diet, Otitis externa, Symptoms intensity decrease, Antiinflammatory and antioxidant activities

## Abstract

The aim of this evaluation study was to assess the possible role of a specific nutraceutical diet in relieving main clinical symptoms of chronic bilateral otitis externa (occlusion of ear canal, erythema, discharge quantity, and odor) in 30 adult dogs. Thirty dogs of different breeds (mean age ± SEM; 6.03 ± 0.15 years and mean weight ± SEM; 32.01 ± 1.17 Kg; 53.3 % males, 46.6 % females) with evident chronic clinical otitis symptoms were equally divided and randomly assigned to receive either the nutraceutical diet (ND group) or a standard diet (SD group) over a period of 90 days. In all cases a topical pharmacological treatment was given. The nutraceutical diet, also endowed with anti-inflammatory and antioxidant activities, significantly decreased the mean score intensity of all symptoms after 90 days of intervention (*P* < 0.0001) with the exception of *Malassezia pachydermatis* infection which was only slightly reduced. Our investigation is one of the few evidence-based results where a commercial nutraceutical diet has been proven effective, in combination with drugs, in relieving otitis externa-related symptoms. This study opens new insights into otitis externa clinical management providing evidence of efficacy of a combined therapy with drugs and a specific nutraceutical diet.

## Text

Otitis externa is supposed to affect 4 out of 1,000 persons annually in USA (Osguthorpe and Nielsen 2006). Its chronic expression affects 3–5 % of the same population (Agius et al. 1992; Daneshrad et al. 2002; Hannley et al. 2000; Sood et al. 2002) whereas the acute one is unilateral in 90 % of cases and affects 7 to 12 years aged people declining after 50 years. Further, the acute otitis externa is often associated with local trauma, hearing aids, swimming, warmer temperatures, high humidity and hearing protector use (Beers and Abramo 2004). Otitis externa is commonly due to bacterial or occasionally fungal infections (Sander 2001) following an increased ceruminal pH level (Halpern et al. 1999), which enhances the microbial growth (Beers and Abramo 2004; Daneshrad et al. 2002; Sander 2001; Tsikoudas et al. 2002), and/or an insufficient amount of earwax (Beers and Abramo 2004; Sander 2001). Early clinical symptoms are pruritus, erythema and pain. As the disease proceeds, the erythema increases and is followed by edema and otorrhea. If untreated, the pain becomes intense, the lumen of the ear canal gets obstructed and the conductive hearing loss might occur (Beers and Abramo 2004; Daneshrad et al. 2002; Sander 2001).

Otitis externa is also one of the more frustrating disease affecting pets (Pietschmann et al. 2013). Its clinical evolution can be summarized in three phases: 1) acute inflammation and edema, 2) chronic inflammation (glandular changes, fibrosis and scarring) and 3) progressive stenosis and occlusion of the ear canal (Logas 1994; Roth 1988). Calcification and even ossification of cartilage might also occur as well as otitis media and aural cholesteatoma (Logas 1994). Chronic processes, as a consequence, enhance bacteria moltiplication, such as *Pseudomonas spp,* with secondly induced lesions (McKeever and Torres 1997; Roth 1988). It is generally recognized that cleaning and drying the ear canal can reduce inflammation and resolve secondary infections (Rosychuk 1994). However, antimicrobials (Polimixin B, Enrofloxacin, Orbifloxacina, rifaximin, Gentamicin, etc.) and antimycotics (Miconazole, Clotrimazole, Posaconazole, etc.) remain the gold standard against most of pathogens (*Staphylococcus spp, Pseudomonas aeruginosa, Escherichia coli*, *Proteus mirabilis* and *Malassezia pachydermatis*) (Engelen et al. 2010; Engelen and Anthonissens 2000; Peano et al. 2012; Rougier et al. 2005; Studdert and Hughes 1991). *Malassezia pachydermatis* has been identified as the most common yeast organism present in ears of dogs affected by otitis externa (Cole et al. 2007; Crespo et al. 2002). This yeast colonizes the ear canal surface and is usually found adherent to clumps of exfoliated squamous epithelial cells (Porter 2011). It can be rapidly identified by microscopic examination and normally should not exceed 10 organisms per high-power field (Cowell et al. 2008).

The aim of this clinical evaluation was to observe the effect of a commercial nutraceutical diet, also endowed with anti-inflammatory and antioxidant activities, as an adjuvant in pharmacological treatment of dogs affected by chronic otitis externa in order to improve the intensity of its clinical signs as well as the presence of *Malassezia pachydermatis*. The antiinflammatory and antioxidant activities of the proposed diet have to ascribed to the presence of the pool of fish hydrolyzed proteins, rice carbohydrates, *Melaleuca alternifolia, Tilia cordata, Allium sativum L, Rosa canina L.,* Zinc and a well balanced Omega3:6 ratio (1:0.8).

In this regard, Tea tree oil (TTO) of *Melaleuca alternifolia* has been widely used as antimicrobial (Carson et al. 2006; Mikus et al. 2000) and anti-inflammatory phytotherapic coumpound [(reduction of Tumor necrosis factor-α, Interferon-γ, Interleukin-2] (Baldissera et al. 2014) for the presence of terpinen-4-ol and 1.8-cineole (Caldefie-Chezet et al. 2006; Dalwai et al. 2014; de Campos Rasteiro et al. 2014; Furneri et al. 2006; Greay et al. 2010; Hammer 2015; Ireland et al. 2012; Mantil et al. 2015; Nogueira et al. 2014). TTO is also known to exert antioxidant effects on human peripheral blood mononuclear cells by reducing reactive oxigen species production and IL-2 secretion in T lymphocytes, and increasing the secretion of the anti-inflammatory cytokines such as Interleukin-4 and Interleukin-10 (Caldefie-Chezet et al. 2006). Several human studies have also evidenced the benificial effect of TTO in experimentally induced skin reactions (nickel- or histamine-induced contact hypersensitivity) (Khalil et al. 2004; Koh et al. 2002; Pearce et al. 2005; Wallengren 2011).

Anti-inflammatory and antioxidant activities have been also ascribed to flowers, bracts and leaves of *Tilia cordata,* usually known as lime tree (Russo et al. 2000; Scherl et al. 2012; Toker et al. 2001)*.* Antioxidant (Banerjee et al. 2001, 2002; Fanelli et al. 1998; Lau 2001; Lin et al. 1996; Maslin et al. 1997; Prasad et al. 1996), antimicrobial (Dini et al. 2011; Jonkers et al. 1999; Karuppiah and Rajaram 2012; Wills 1956), anti-protozoal (An et al. 2009; Perez et al. 1994; Watson 1996), antifungal (Adetumbi et al. 1986; Ghannoum 1988; Shams-Ghahfarokhi et al. 2006; Szymona 1952), antiviral (Guo et al. 1993; Tsai et al. 1985; Weber et al. 1992), hypotensive (Chaupis-Meza et al. 2014; Majewski 2014; Rashid and Khan 1985; Reinhart et al. 2008; Ried et al. 2010; Sobenin et al. 2009; Stabler et al. 2012), cardioprotective (Allison et al. 2012; Ashraf et al. 2013; Bordia et al. 1998; Sumiyoshi and Wargovich 1990) and anti-tumor (Amagase and Milner 1993; Capasso 2013; Lin et al. 2002; Sumiyoshi and Wargovich 1990; Tadi et al. 1991a, b; Tsubura et al. 2011; Wallace et al. 2013; Wang et al. 2012) effects were observed for *Allium sativum L.,* commonly known as garlic, due to the presence of biologically active substances such as allicin, ajoene and diallyl trisulfide. *Rosa canina L.* is a plant whose berries are endowed with antioxidant, anti-inflammatory, immunomodulating and antimicrobial activity due to the presence of phenolic acids, proanthocyanidins, tannins, flavonoids, unsaturated and polyunsaturated fatty acids, phospholipids, minerals, galactolipids, carotenoids and triterpenes (Chrubasik et al. 2008; Sadigh-Eteghad et al. 2011). This plant exerts a specific anti-inflammatory activity (Jager et al. 2007, 2008; Larsen et al. 2003; Lattanzio et al. 2011; Wenzig et al. 2008), some immunomodulatory and antioxidant activities (Davitashvili et al. 2010; Sadigh-Eteghad et al. 2011; Sies et al. 1992; Takashima et al. 2012; Tumbas et al. 2012), and antimicrobial effects (Shiota et al. 2000). Additional activities ascribed to this plant are antiulcerogenic and probiotic (Deliorman Orhan et al. 2007; Gurbuz et al. 2003; Johansson et al. 1998), hypoglycemic (Ninomiya et al. 2007), antimutagenic and anticancerogenic (Trovato et al. 1996).

Immunomodulatory activities have also been ascribed to zinc, whose deficiency affects innate and adaptive immunity, exacerbates inflammation (Bonaventura et al. 2014) and is closely related to skin desease and wound healing (Colombini 1999) since its absolute or relative deficiency can cause the onset of canine zinc-responsive dermatosis (Hensel 2010).

An optimal balance of the omega 3:6 fatty acids ratio in the food is considered a fundamental requirement for tissue to improve homeostasis and contrast the inflammatory processes. More in details, n-3 polyunsaturated fatty acids, usually found in fish oil, such as eicosapentaenoic acid (EPA) and docosahexaenoic acid (DHA), are known to decrease the production of proinflammatory mediators and inhibit natural killer cell activity (Kelley et al. 1999). In addition, the n-6 polyunsaturated fatty acid gamma-linolenic acid (GLA) and EPA are endowed with specific antiinflammatory activity (DeLuca et al. 1999).

Based on such considerations, we performed a randomized placebo-controlled clinical evaluation on 30 dogs with evident chronic clinical otitis symptoms such as occlusion of ear canal, erythema, discharge quantity, and odor.

## Materials and methods

### The animals

Thirty adult dogs of different breeds (mean age ± SEM; 6.03 ± 0.15 years and mean weight ± SEM; 32.01 ± 1.17 Kg; 53.3 % males, 46.6 % females) with evident chronic clinical otitis symptoms were randomly divided and assigned to receive either the specific diet (treatment group, *n* = 15) or the placebo (control group, *n* = 15) once a day for 90 days, accordingly with the following manifacture’s table (Table 1). In addition, all dogs were also pharmacologically treated with a topic product (OTOMAX, Schering-Plough, Kenilworth, NJ, USA) 8 drops a day for 7 days.

**Table 1:** Daily amount of dietary supplement suggested by the manifacturer

Daily ratio	
Weight (Kg)	Amount (g)
1 – 10	30 – 180
11 – 20	190 – 300
21 – 35	310 – 455
36 – 50	465 – 595

### The diets

The two diets were based on the same receipt and completely fulfil the recommendations for proteins, carbohydrates and fats content in order to obtain a complete food for a daily ration in dog (as reported in Nutritional Guidelines for complete and complementary pet food for cats and dogs by The European Pet Food Industry Federation). In particular, the two foods reported similar analytical composition in nutrients (24 % of crude protein, 12 % of crude oils and fats, 3.7 %, of crude fibre 5 % of crude ash, 9 % of moisture) and, as a consequence, similar Metabolised Energy (ME) of 3.477 kcal/kg corresponding to 14.6 MJ/kg. Both foods are commercially available and in the form of kibbles industrially produced with extrusion technique. The specific nutraceutical diet was composed by two mixed components: kibbles, included in the ideal percentage of 93–94 % in weight, and cold-pressed microcapsules at the 6–7 % in weight of complete food (European patent n.EP 2526781). Overall nutrient profile of the product was obtained by the sum of a first nutrient profile of the kibbles, for feeding purpose, and a second nutrient profile of the microcapsules for both nutrient and therapeutic purposes. Microcapsules were composed of 60–80 % of hydrolyzed proteins (of fish and vegetable origin), 20–40 % of minerals, used as glidants, and therapeutical substances (*Melaleuca alternifolia*, 0.00343 %*, Tilia platyphyllos scapoli et cordata,* 0.0147 %*, Allium sativum L.,* 0.0245 %*, Rosa canina L.,* 0.098 %, and Zinc*,* 0.00479 %).

### Malassezia pachydermatis determination

**Fig. 1:**
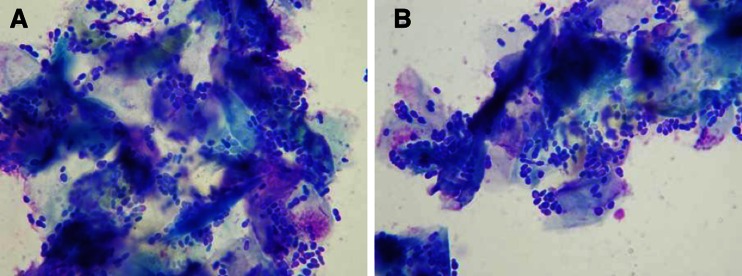
Microscopic image of *Malassezia* presence. Microscope image (100X) highlighting the presence of several *Malassezia pachydermatis* organisms along with epithelial cells at different mature stages.

A small-tip cotton swab was inserted into the external ear canal removing some exudate. The swab was then rolled along a microscope slide with the sequence number. The slides were dried and stained with modified Wright’s stain, and evaluated microscopically (Cole et al. 2007) with an Olympus 60BX polarized light microscope (New York Microscope Company Inc, Hicksville, NY, USA). *Malassezia pachydermatis* organisms were identified morphologically. The sample was considered pathological if the average of identified yeasts resulted more than 10 per high-power field (HPF) in several fields, (Fig. 1) (Cowell et al. 2008). The precedure was performed before intervention (time 0); after 30 days (time 30); after 60 days (time 60) and at the end of intervention (time 90).

### Clinical evaluation and scoring system

Dogs received veterinary inspections, before intervention (time 0); after 30 days (time 30); after 60 days (time 60) and at the end of intervention (time 90).

Operative procedures and animal care were performed in compliance with the national and international regulations (Italian regulation D.L.vo 116/1992 and European Union regulation 86/609/EC). The protocol was examined and approved prior to the beginning of the study by the Veterinary Ethical Review Committee. The recommendations of the ARRIVE guidelines in animal research were also consulted and considered (Kilkenny et al. 2012).

Immediately before treatment, and at the end, the condition of the ears was assessed always by the same operator and scored for the following clinical signs (Hawkins et al. 2010):

- Occlusion of ear canal (0–3); 0 = normal, 1 = occluded (but possible to insert a 6 mm otoscope (Operative Otoscope, HEINE Optotechnik, Herrsching, Germany) nozzle into the vertical ear canal), 2 = occluded (but possible to insert a 4 mm nozzle), 3 = occluded (not possible to insert a 4 mm nozzle).
- Erythema (0–3); 0 = normal, 1 = mild, 2 = moderate, 3 = severe.
- Discharge quantity (0–3); 0 = absent, 1 = slight, 2 = moderate, 3 = profuse.
- Odor (0–3); 0 = absent, 1 = mild, 2 = moderate, 3 = intense.

### Statistical analysis

Data were analyzed using GraphPad Prism 6 software (GraphPad Software, Inc., La Jolla, CA, USA). All data are presented as the means ± standard error of the mean and were first checked for normality test using the D’Agostino-Pearson normality test. Differences in occlusion of the ear, erythema, discharge quantity and odor score between the two supplements at the end of treatment versus baseline for each ear were analyzed using a two-way analysis of variance (ANOVA) followed by Sidak’s multiple comparisons test. A *p* < 0.05 was considered significant.

## Results

Following clinical and cytological evaluation 28 out of 30 dogs presented an excessive amount of ear wax related to *Malassezia pachydermatis* infection. Only 2 out of 30 dogs reported an additional bacterial presence (either cocci or bacilli), therefore we considered such condition as not worth of clinical monitoring.

**Fig. 2:**
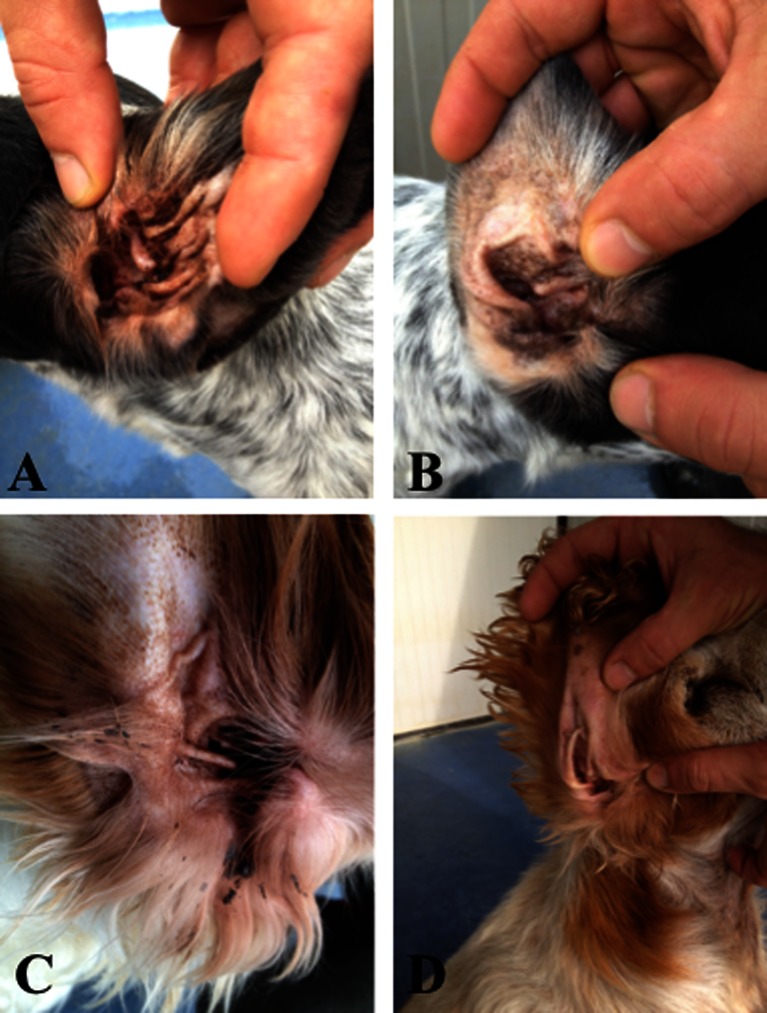
Ears improvement after 90 days of evaluation with specific nutraceutical diet, (**a**-**c**) ears before the evaluation (time = 0), (**b**-**d**) ears at the end of the evaluation (time = 90)

No adverse effects, such as cutaneous atrophy, secondary infections (Muller et al. 2001), increased licking (Bensignor and Olivry 2005), occasional skin itching or burning (Caffier et al. 2007) and hearing loss (Mason et al. 2013) were reported by the owners or noted on otoscopic examinations with any treatment, and all dogs completed the 90-day evaluation period. In Fig. 2, the overall improvement of dogs hears before and at the end of the 90-days evaluation is shown (Fig. 2).

**Fig. 3:**
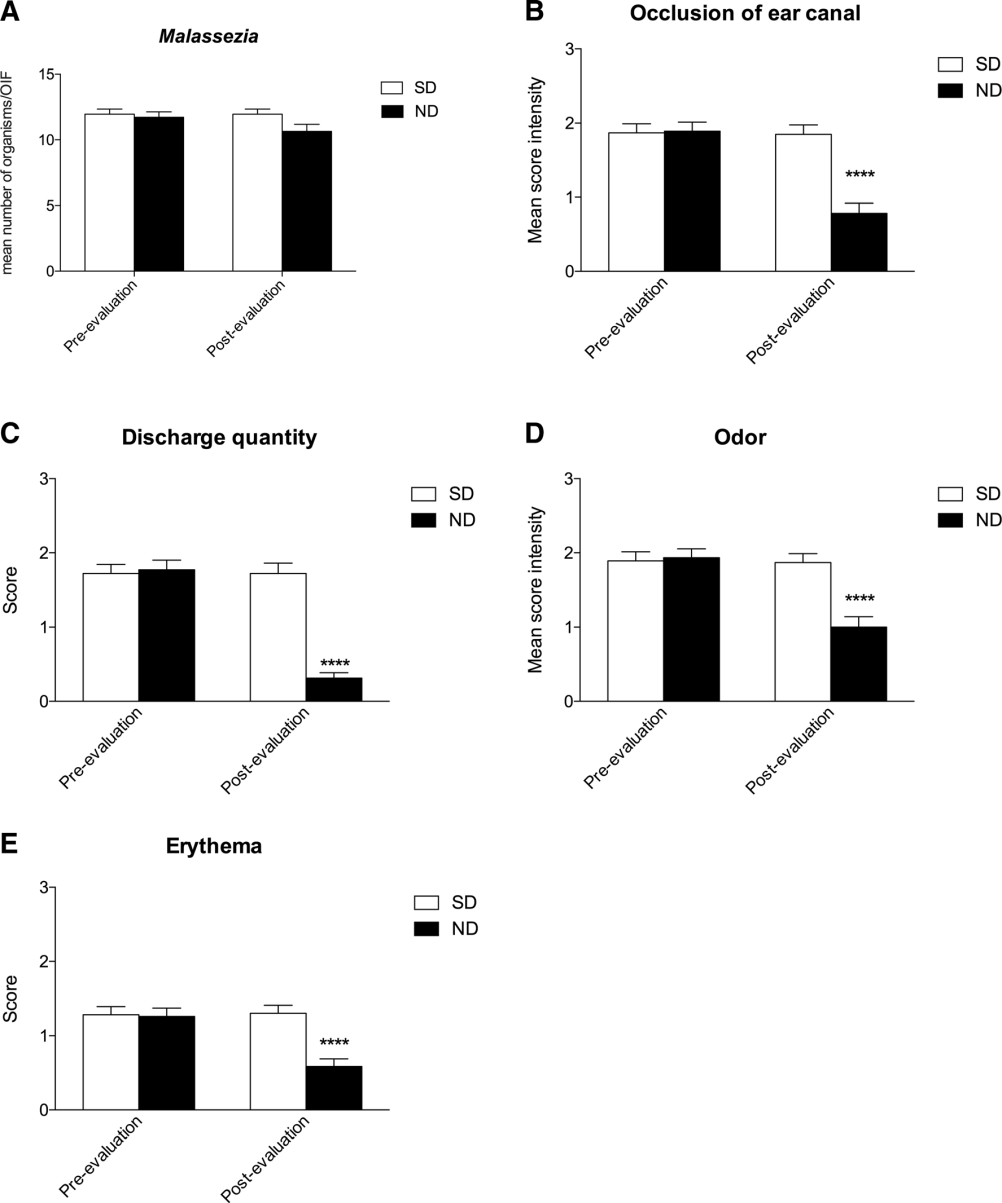
Graphical representations of symptoms trend during the evaluation. (**a**) Mean *Malassezia* organisms in OIF before and after 90 days of evaluation for SD and ND group, organisms resulted slightly decreased in ND group; (**b**) mean occlusion of ear canal score before and after 90 days of evaluation for SD and ND group, a significant decrease (****P < 0.0001) was observed in ND group; (**c**) mean discharge quantity score before and after 90 days of evaluation for SD and ND group, a significant decrease (****P < 0.0001) was observed in ND group t; (**d**) mean odor score before and after 90 days of evaluation for SD and ND group, a significant decrease (****P < 0.0001) was observed in ND group; (**e**) mean erythema score before and after 90 days of evaluation for SD and ND group, a significant decrease (****P < 0.0001) was observed in ND group

The nutraceutical diet significantly decreased dog’s ear canal occlusion, erythema, odor and mucus discharge scores after 90 days of evaluation, if compared with baseline while the mean number of *Malassezia pachydermatis* organisms slightly decresed (Fig. 3).

After 90-days of evaluation *Malassezia pachydermatis* organisms decreased from a baseline value of 5.32 ± 0.4 to 4.2 ± 0.3 in the ND group and from a baseline value of 5.4 ± 0.4 to 5.4 ± 0.3 in the SD group (Fig. 3a).

Dogs ear canal occlusion resulted decresead from a baseline value of 1.87 ± 0.1 to 1.84 ± 0.1 in the SD group and from a baseline value of 1.89 ± 0.1 to 0.78 ± 0.1 in the ND group (Fig. 3b, ****P < 0.0001).

As to discharge quantity, the scores decreased from a baseline value of 1.72 ± 0.1 to 1.74 ± 0.1 in the SD group and from a baseline value of 1.77 ± 0.1 to 0.31 ± 0.07 in the ND group (Fig. 3c, ****P < 0.0001). Also odor scores resulted decreased after 90-days of evaluation, with respect to the baseline.

More in details, the scores decreased from a baseline value of 1.89 ± 0.1 to 1.87 ± 0.1 in the SD group and from a baseline value of 1.91 ± 0.1 to 1.0 ± 0.1 in the ND group (Fig. 3d, ****P < 0.0001).

Finally, erythema dicreased from a baseline value of 1.28 ± 0.1 to 1.30 in the SD group and from a baseline value of 1.26 ± 0.1 to 0.58 ± 0.1 in the ND group (Fig. 3e, ****P < 0.0001).

## Discussion

Dogs affected by chronic, recurrent otitis externa are considered one of the most frustrating pathologies of daily veterinary clinical practice (Rosser 2004).

In this study, we use a specific nutraceutical diet, based on a combination of fish hydrolyzed proteins, rice carbohydrates, *Melaleuca alternifolia, Tilia cordata, Allium sativum L, Rosa canina L.,* Zinc and a Omega3/6 (1:0.8 ratio), as an adiuvant approach for the clinical management of canine otitis externa.

We observed a significant and encouraging reduction of the main symptoms of otitis externa - as the external ear canal occlusion, erythema, odor and mucus discharge - in enrolled dogs fed the nutraceutical diet if compared to those who received the standard diet. To this regard, we recently described the efficacy of a similar diet in relieving some otitis externa clinical symptoms, such as malodor, shaking, pus presence, earwax, itch, edema, blood presence, auricular function and auricular flush, in 107 dogs after 30 days of evaluation (Di Cerbo et al. 2014).

Our results appear in agreement with those observed by Sarrell et al. that compared the effectiveness of a naturopathic herbal extract, containing also *Allium sativum,* with anaesthetic ear drops in the management of ear pain associated with acute otitis media (Sarrell et al. 2001). Specifically, 61 out of 103 children treated with naturopathic herbal extract had an overall improvement in ear pain score due to analgesic, anti-inflammatory, anti-occlusive effects and anti-infective properties of the naturopathic product.

Here, we evidenced that the specific nutraceutical diet was also highly tolerated throughout the whole evaluation period as no adverse effects were observed in all dogs completing the study. In addition, we observed that most of clinical symptoms were substantially halved. These intersting occurences strongly encourage the use of the nutraceutical diets, endowed with anti-inflammatory and antioxidant acitivities, as valid and safe support to the conventional pharmacological therapy for dogs affected by chronic otits externa.

With regard to comorbidity of *Malassezia* infection, the addition of zinc in our diet was based on previous *in vitro* and *in vivo* studies, which highlighted its role in reducing yeasts number (DeAngelis et al. 2005; Mendelsohn et al. 2005). However, clinically apparent yeast presence seemed unvaried in the SD group. Althought our study showed a slightly reduction in the number of *Malassezia pachydermatis* organisms in ND group, it is reasonable to hypothesize a possible synergistic action of this antiinflammatory and antioxidant diet with antibiotic therapy. In this regard, therapy usually is topically applied for a reduced time, in order to avoid an antibiotic resistance phenomenon. It is noteworthy that an increased risk of antibiotic resistance may occur after a routine topical antibiotic administration in the treatment of otitis externa (Voget et al. 2012). In this respect, the anti-inflammatory and antioxidant effects of a diet could likely reduce the needing and the frequency of local antibiotic administration and contribute to avoid the emergence of drug resistence.

The results achieved in this study, concerning ear canal occlusion and erythema, are in agreement with those observed by Sarrell et al. that compared the effectiveness of a naturopathic herbal extract, containing also *Allium sativum,* with anaesthetic ear drops in the management of ear pain associated with acute otitis media (Sarrell et al. 2001). Specifically, the authors reported that 61 out of 103 children, belonging to the naturopathic herbal extract-treated group, had an overall improvement in ear pain score due to analgesic, antiinflammatory, anti-occlusive effects and anti-infective properties of the naturopathic product.

Many studies, regarding both dog and human, claim for nutraceutical administration benefits in otitis externa. Our investigation further outlines the quick symptoms relieving of otitis externa by means of a commercially available nutraceutical diet.

To the best of our knowledge this is the first report of a veterinary clinical evaluation concerning a anti-inflammatory and antioxidant diet effect on dogs affected by chronic otitis externa. Although further studies with a larger sample and time of observation are needed these results can be considered very promising in light of a possible traslation on the human side.
